# Split-Brain Babies? Differences in Representation of Bilaterally and Unilaterally Presented Visual Stimuli in Infancy

**DOI:** 10.3389/fpsyg.2018.02758

**Published:** 2019-02-04

**Authors:** Kimberly M. Scott

**Affiliations:** Early Childhood Cognition Lab, Department of Brain and Cognitive Sciences, Massachusetts Institute of Technology, Cambridge, MA, United States

**Keywords:** interhemispheric integration, corpus callosum, split brain, approximate number system, infant perception, looking time

## Abstract

Information needed for perception and action is often distributed across the two hemispheres of the human brain. During development, representations lateralized due to topographic sensory maps may be available independently before they can be integrated across hemispheres. These studies (total *N* = 211) investigate visual interhemispheric integration in two domains in infancy. In Experiment 1, infants (8–14 months) showed stronger evidence of representing the equality of two shapes when the shapes were presented in the same visual hemifield. In Experiments 2–4, infants (10–19 months) showed evidence of greater familiarization when shown 16 dots in one hemifield than when shown 8 dots in each hemifield. The possibility that interhemispheric integration poses an unusually late-resolved challenge in infant vision is discussed.

## Introduction

The distribution of computation across the human brain carries with it a mandate to communicate across functionally distinct regions. When communication between specialized regions is impaired, localized abilities and representations become sequestered. The consequences are especially dramatic in the case of callosotomy, or “split-brain,” patients, in whom the corpus callosum has been surgically severed to control epileptic seizures. In these patients, each hemisphere processes and acts upon the information available to it – for instance, the right hemisphere has access only to the left visual hemifield. Despite some subcortical transfer of information, and compensatory strategies that allow patients to go about their daily lives with surprisingly minimal impairment, careful testing reveals an experience that is in many ways like that of two individuals sharing one body (for review, see Zaidel, 1994). The segregation of abilities and perception in split-brain patients—when established interhemispheric connections have been severed—raises the intriguing possibility of a similar experience in early childhood when those connections are first developing.

We expect that interhemispheric integration may pose a particularly long-lasting challenge for young children due to the protracted maturation of the corpus callosum, the bundle of approximately 200 million axons that serves as the only direct connection between the left and right cortical hemispheres (for a review, see Paul, 2011). In particular, myelination—the process of depositing axonal insulation to allow neural signals to travel faster—begins 4–6 months after birth and proceeds gradually through at least the first two decades of life (Barkovich and Kjos, 1988; Giedd et al., 1999; Lebel et al., 2008). Corroborating the importance of myelination in information transfer, children with reduced myelination due to phenylketonuria exhibit selective difficulties with interhemispheric communication, (Banich et al., 2000). The increasing speed of signal transmission with myelination is observable through adolescence as a decrease in the delay between ipsilateral and contralateral event-related potentials following unilateral stimulation (Salamy, 1978).

Concurrent changes in behavior on tasks that involve interhemispheric processing suggest that this protracted development has functional consequences throughout early childhood. For instance, hemispheric specialization for language in the left hemisphere is directly observable via lateralized visual presentation in preschoolers: children make fewer accurate and more confabulatory responses following presentations to the right hemisphere (Joseph et al., 1984; Liégeois and de Schonen, 2002). Through at least the early elementary years, improved interhemispheric communication is marked by progress in the transfer of tactile information (Galin et al., 1979) and in bimanual coordination (O’Leary, 1980; Greiner and Fitzgerald, 1992; Fagard et al., 2001). Similar to callosal agenesis patients, in whom the corpus callosum never develops at all, children up to 6 years old fail to transfer a manual task they learned in one hemisphere to the other (Chicoine et al., 2000) or to exhibit bimanual cost, an index of interhemispheric inhibition (Franz and Fahey, 2007). While findings on the degree of impairment are mixed, and comparison among results is complicated by task differences, it is clear that it takes at least through middle childhood to reach an adult-like state of interhemispheric communication.

In infancy, deficits in interhemispheric communication are potentially more profound, although findings are mixed and failures potentially complicated by heavy task demands of learning visual rules via operant conditioning. For instance, although infants appear to partially transfer knowledge of a visual rule learned in one hemisphere to the other by 4–6 months (de Schonen and Bry, 1987), evidence for immediate comparison of shapes presented in opposite hemifields has been found only much later, at 2 years of age (Liégeois et al., 2000). In line with these findings, at 6 months of age, infants are able to transfer information about faces between hemispheres during learning: the right hemisphere can “recognize” faces that were presented to the left. However, this transfer does not allow the right hemisphere to discriminate faces at the time they are presented to the left hemisphere (Adibpour et al., 2018).

The present study addresses the possibility that infants may indeed represent some concepts individually in each hemisphere substantially before they are able to integrate this information across hemispheres during perception. In contrast to children’s difficulty coordinating physical actions or transferring learned rules from one hemisphere to the other, differences in perceptual integration would imply that early phenomenological experience is not characterized by the spatial unity adults report. To assess what infants perceive during bilateral vs. unilateral visual stimulation, we developed a protocol using a lateralized familiarization procedure followed by free-viewing looking-time tests. This is, in effect, an infant adaptation of the protocols used to measure split-brain patients’ ability to integrate or compare visual information across the two visual hemifields (Corballis, 1994; Seymour et al., 1994; Sperry et al., 1969). We hypothesized that infants would behave as if familiarized with the stimuli in each visual hemifield separately, leading to differences between bilateral and unilateral presentation of the same images. We focused on the visual domain because of the brain’s reliance on retinotopic mappings throughout both primary sensory cortex and higher-level visual association areas. This allows us presentation of a visual stimulus to one hemisphere of the brain simply by ensuring it appears in the opposite visual hemifield. Visual adaptation and aftereffects—signatures of retinotopic neural responses—suggest that even high-level visual concepts including approximate number (Ross and Burr, 2010) and facial identity (MacLin et al., 1996; Leopold et al., 2001) are represented by spatially localized detectors.

## Experiment 1: Is a Square on the Left the Same Shape as a Square on the Right?

We first sought to confirm infants’ difficulty in comparing shapes from opposite visual hemifields, reported by Liégeois et al. (2000). In that study, children were conditioned to look up or down based on whether two shapes matched. Children under 24 months succeeded in learning this rule only when the two shapes were presented in a single visual hemifield. However, potential alternative explanations remain for the failure: the effect was specific to face stimuli, the bilateral presentations were presented foveally near a fixation light, and learning separate motor responses to both “same” and “different” pairs is difficult for children despite being able to represent abstract sameness (Hochmann et al., 2016). In the present experiment, we tested a procedure intended to measure infants’ perception of bilaterally presented stimuli without requiring rule learning or an explicit motor response. Given uncertainty about the potential age at which to expect this ability to emerge, we tested infants aged 8–14 months, substantially under the age at which Liégeois et al. (2000) demonstrated success in their conditioning task.

We familiarized infants with matching shapes either unilaterally or bilaterally by very briefly presenting matching pairs of shapes while infants were looking at a small video. The short presentation duration prevented infants from being able to simply saccade to the shapes. This familiarization period was designed to affect their preference for matching shapes, measured before and after familiarization. If infants integrate information across hemifields, they should perceive two matching shapes regardless of whether the shapes are presented unilaterally or bilaterally, but if they do not, they should perceive the match only when the shapes are presented unilaterally.

### Methods

#### Availability of Materials and Preregistration of Data Analysis

The experiments described here were conducted starting in 2013, before our lab began to preregister studies. After completing data collection and some preliminary analysis, a final analysis plan was registered on the Open Science Framework to disclose all prior pilot data collected and analysis completed, minimize analytic degrees of freedom, and standardize procedures across studies. All blind coding and participant-level inclusion decisions were finalized before proceeding with analysis of the relevant data. When available, data collected after the planned sample size was reached were included in analysis. Sample sizes were originally set in advance of data collection (based on convention for Experiments 1–3; based on power analysis using early analysis of Experiment 2 for Experiment 4) and stopping was not influenced by preliminary results. However, subjects initially excluded (e.g., due to a single invalid looking-time trial) were sometimes included because the final analysis used linear mixed-effects models robust to missing data. The registration, stimuli, presentation and analysis code, and results of video coding are available on the Open Science Framework at https://osf.io/5fds4/.

#### Participants

Infant participants were recruited at the Boston Children’s Museum and parents provided informed consent to participate. Although detailed demographic information is not available, most participants were from white or Asian, upper-middle-class backgrounds and had a highly educated stay-at-home parent. We do not routinely collect information about gestational age at birth or any medical history beyond what parents spontaneously report; there were no exclusion criteria besides those listed below. Each participant’s age in months was calculated as the age in days times 12 divided by 365. Forty-nine infants (20 female) between 8 and 14 months of age (mean age 11.0 months) participated in this study. An additional 34 infants were excluded due to failure to complete the study (*n* = 16), experimenter error (*n* = 7), fussiness (*n* = 6), inattention (*n* = 4), or interference from a sibling (*n* = 1).

#### Procedure

The protocol was approved by the MIT Committee on the Use of Humans as Experimental Subjects. Each child sat on a parent’s lap for the duration of the study, 1.5 m from a large monitor (80 cm × 30 cm) used to display all stimuli. Subjects were video-recorded using a camera positioned directly above the monitor. The experimenter was positioned behind the monitor, hidden from the view of the infant, and monitored the child using a webcam positioned above the monitor while controlling the progression of the study using Psychtoolbox extensions (Brainard, 1997) in MATLAB (Natick, MA, United States).

The procedure consisted of (1) a baseline test of looking time to matching and non-matching pairs of shapes; (2) a familiarization period showing only matching shapes; and (3) a final test of looking time to matching and non-matching pairs of shapes. Prior to the familiarization period, there was a short break (15–60 s) for parent debriefing; prior to the final test, there was another short break (approximately 15 s) as parents were asked to close their eyes. The entire study lasted about 5 min. Infants were assigned to one of three familiarization conditions: bilateral (n = 16), unilateral-peripheral (n = 16), or unilateral-distance (n = 17).

##### Baseline looking time test

**FIGURE 1:**
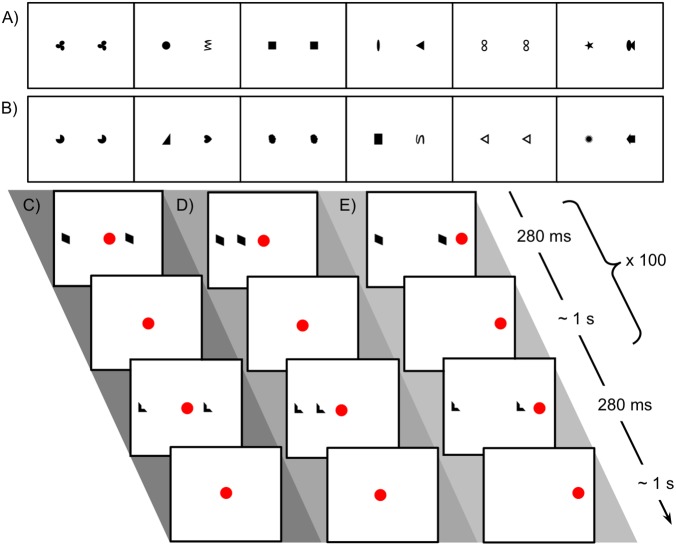
Stimuli used in Experiment 1 for looking time tests and familiarization procedure. **(A)** Sequence of matching and non-matching shape pairs used for looking time tests, test set A. Images are shown in the order presented (left to right) for the counterbalancing condition in which a matching pair was shown first. **(B)** Test set B. **(C)** Schematic representation of procedure for familiarization with matching shapes, bilateral condition. The central red dot indicates the position of the fixation video. The side of the more distant shape was counterbalanced. **(D)** Unilateral-peripheral condition familiarization with shapes presented in a single hemifield but at the same distances from fixation as in the bilateral condition. The right/left position of the shapes was counterbalanced. **(E)** Unilateral-distance condition familiarization, with shapes presented in a single hemifield but at the same distance from each other as in the bilateral condition. The red dot to the right indicates the position of the fixation video; fixation position on left/right was counterbalanced.

We first measured each infant’s initial preference for pairs of matching vs. non-matching shapes. Each test consisted of six trials, alternating between matching and non-matching pairs of shapes. The set of images used in the test, A or B (shown in Figure 1A,B), was counterbalanced. The order of presentation was counterbalanced such that half of the children saw a matching pair first (order 1, 2, 3, 4, 5, 6) and half saw a non-matching pair first (in order 2, 1, 4, 3, 6, 5). To avoid inadvertent bias, parents were asked to close their eyes during the test phase and not to talk. Before each looking time trial, the child’s attention was drawn back to the screen with a continuous chime and a fixation video, and the experimenter waited for the child to be looking before triggering the next image to be displayed. The experimenter ended the trial once the child looked away for at least 1–2 s. Experimenters were not blind to condition or age.

##### Lateralized familiarization: how stimuli were presented unilaterally and bilaterally

During familiarization, infants saw only pairs of matching shapes, presented either unilaterally or bilaterally. Images of matching shapes were presented briefly while the infant was looking at a small “fixation video,” as shown in Figure 1C–E, which looped continuously at a fixed position on the screen. The experimenter triggered brief presentations of exemplars of matching shapes at a maximum rate of about 1 per second, only triggering a presentation when the infant was looking at the fixation video. This allowed presentation in a known hemifield (with deviation only due to saccades already planned or in progress at the time of stimulus presentation): if the infant was looking at a fixation video on the left of the screen, for instance, the image would appear in the right visual hemifield and be processed in the left hemisphere. Note that the infant’s current fixation point (not head position or centering with respect to stimuli) is what determines the split between processing in the right and left hemispheres. Exemplars were presented for 280 ms following de Schonen and Bry (1987), well under the approximate expected saccadic latency of 400 ms (Irving et al., 2006). Because each image was presented so briefly, even if the infant initiated a saccade as soon as it appeared, the shape would be gone by the time his or her gaze arrived at its location. Naturally jittered manual timing of stimulus presentation ensured that infants did not learn to “anticipate” a regularly timed presentation and saccade in advance to “catch” the image when it appeared. Exemplar images were not masked, since continued visual processing of the images via aftereffects would not affect lateralization.

A vertical swatch occupying the central two degrees of visual angle around the fixation video (approximately 6 cm) was held blank to avoid potential bilateral processing of central stimuli, although evidence for foveal overlap is disputed (Lavidor and Walsh, 2004). At least 100 exemplar images were presented to each child; the experimenter continued the protocol after reaching 100 until the child looked away from the screen or approximately 120 presentations were reached. The same exemplars were presented across different conditions by varying the relative position of the fixation video. The presentation order of 73 available exemplars was randomly permuted per child, and the set of images repeated in that order, such that each child saw an exemplar image at most twice.

To help keep children’s attention, music was played during familiarization and experimenters could switch between two fixation videos (a colorful spinning ball and a laughing baby). Parents were not asked to close their eyes during this segment, as blinding to test stimulus presentation would be sufficient to avoid influence on the dependent measure.

##### Conditions

In the ‘bilateral’ condition, the images of matching shapes were on opposite sides of the fixation video, one twice as far from fixation as the other (the position of the more distant shape was consistent within subjects and counterbalanced). In the ‘unilateral-peripheral’ condition, both matching shapes were on one side of the central fixation video (side consistent within subjects and counterbalanced), at the same distances from fixation as in the bilateral condition. In the ‘unilateral-distance’ condition, the fixation video was shifted toward one side (consistent within subjects and counterbalanced) of the monitor to accommodate the placement of both shapes on one side of fixation at a distance from each other equal to the distance in the bilateral condition. Pilot data established that a coder blind to condition could correctly identify the location of the fixation video from the child’s gaze as a basic manipulation check.

We predicted that infants would only recognize the identity of the two shapes when they were presented unilaterally, whereas familiarization would not lead to a representation of “matching shapes” when shapes were in opposite hemifields.

##### Final looking time test

Following familiarization, we measured infants’ looking times to matching and non-matching shape pairs, following the same procedure as in the baseline test. Infants who saw image set A at baseline saw image set B at test, and vice versa. Infants who saw a matching pair as the first baseline test image also saw a matching pair as the first final test image. This enabled us to look at whether infants’ attention to matching versus non-matching shapes differed from baseline.

#### Video Coding

To extract looking times from video recordings of participants, a coder blind to experimental condition recorded whether the child was looking to the screen for each video frame during test trials. The coder also recorded any intervals where the child was fussy or the parent peeked at the stimuli or talked, and any times when an external distraction caused the child to look away. Coding was performed using VCode (Hagedorn et al., 2008) and the output processed in MATLAB (code available in OSF project repository). Looking time for each trial was defined as the time from the start of image presentation or the child’s initial look to the image, whichever was later, until the start of the child’s first continuous 1 s lookaway. Looking times were capped at 30 s. Invalid trials were defined as those where looking time did not reach 30 s and there was no continuous 1 s lookaway before the experimenter ended the trial; however, these were included in analysis by defining the looking time as the time until the end of the trial.

#### Inter-Coder Agreement and Data Quality

Across the four experiments described here, 14 videos were coded by a second blind coder for looking time and 11 additionally for fussiness, peeking, talking, and distraction. The mean absolute difference in looking time between coders was 0.52 s (σ = 0.82 across 14 participants). Coders almost always agreed on whether looking time was less than 1 s (99% agreement, Cohen’s kappa = 0.79, *n* = 90 trials) and on whether trials were valid (98% agreement, Cohen’s kappa = 0.74, *n* = 90 trials). Agreement on whether trials contained fussing, distractions, peeking, and talking ranged from 96 to 99% across 72 trials. For an overview of the prevalence of common issues with data collection, for instance invalid trials or parental peeking, see Table 1.

**Table 1:** Percentage of trials and participants exhibiting common data quality issues per experiment.

	Experiment 1		Experiment 2		Experiment 3		Experiment 4	
# Participants		49		46		52		68
Trials/participant		8		6		6		6
Looking time <1 s	4.1	24.5	2.9	13.0	5.1	26.9	7.1	35.3
Fussing during trial end	2.8	18.4	5.1	15.2	5.4	23.1	6.4	26.5
Parent peeks	10.2	28.6	9.8	23.9	1.6	9.6	5.6	17.6
Invalid LT measurement	12.2	61.2	9.1	39.1	1.9	9.6	1.5	5.9
Distraction during trial	0.5	4.1	1.1	6.5	1.3	7.7	0.2	1.5
Not looking at trial start	3.3	24.5	3.3	17.4	6.4	26.9	5.1	29.4


*The white column shows the percentage of *trials* with each issue and the shaded column shows the percentage of *participants* with at least one trial with this issue. In Experiment 1, only the eight trials analyzed are considered. In Experiments 2–4, participants excluded due to lack of at least one usable trial per stimulus type are counted here.*

#### Dependent Measures

The first four trials of each test period (baseline and final) were used in analysis, in order to allow retention of data where one of the last trials was missing due to fussiness. A preference score for non-matching shapes was computed for each subject by dividing the sum of the child’s looking times to non-matching pairs (trials 1 and 3 or 2 and 4) by the sum of the looking times to all four pairs (trials 1–4). Preference scores thus ranged from 0 to 1 with a score of 0.5 indicating equal looking times to matching and non-matching pairs.

A shift in preference toward non-matching shapes was computed for each participant by subtracting the baseline from the final preference score; a positive shift indicated that the preference for non-matching shapes increased over the course of familiarization. Our goal was to assess the impact of familiarization on the child’s preference for matching shapes. If he or she represented this concept, the preference would shift due to familiarity, leading to greater differences between baseline and test measurements in the unilateral conditions.

### Results

**FIGURE 2:**
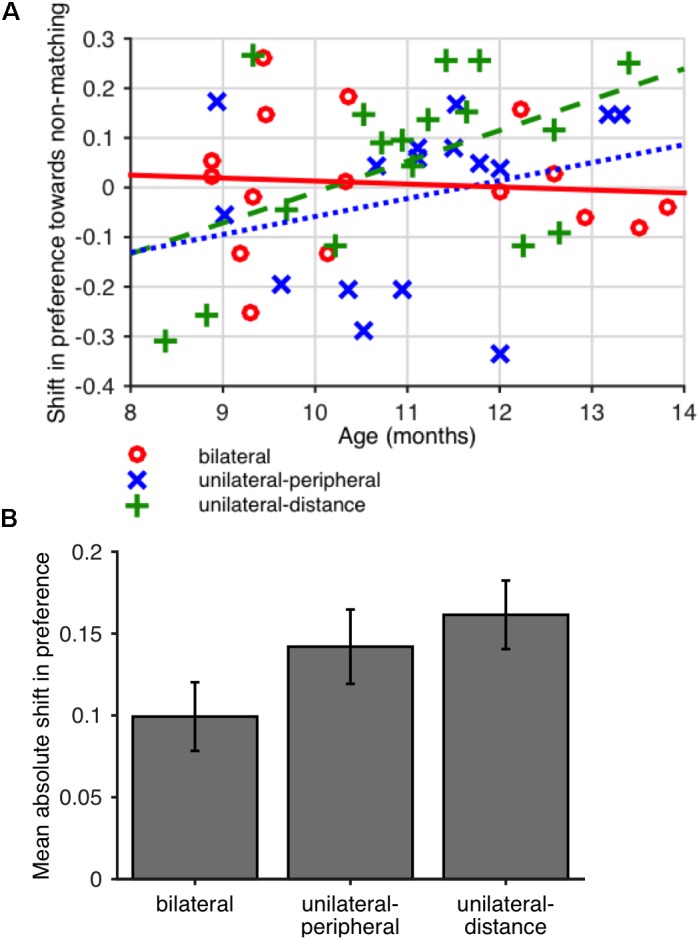
**(A)** Mean absolute value of the difference in preference for non-matching shapes (looking time to non-matching shape pairs divided by total looking time) between baseline and final looking time tests in Experiment 1. Error bars show SEM across participants. **(B)** Difference in preference for non-matching shapes between baseline and final test periods as a function of age in Experiment 1. A positive difference indicates a greater preference for non-matching shapes at final test. Lines (solid red line: bilateral; dotted blue line: unilateral-peripheral; dashed green line: unilateral-distance) show least-squares linear fits for each condition.

Our primary finding in this preliminary study was that the absolute value of the shift in preference was greater in the unilateral conditions (pooled) than in the bilateral condition (Wilcoxon rank sum test, one-tailed, *p* = 0.022; see Figure 2A). This greater change indicated a larger absolute effect of familiarization on preferences in the unilateral conditions, although the direction of the change varied across individuals.

To better understand the nature of the preferences potentially induced by familiarization, we also evaluated the correlation between participants’ age and shift in preference, expecting that the same familiarization period might induce familiarity preferences in younger infants and novelty preferences in older infants. The shift in preference was positively but not significantly correlated with age in the unilateral conditions (unilateral-peripheral: *r* = 0.23, *p* = 0.386; unilateral-distance: *r* = 0.35, *p* = 0.165; Spearman rank correlations) but not in the bilateral condition (*r* = -0.05, *p* = 0.863, Spearman rank correlation). That is, in the unilateral conditions, younger infants showed a slight shift toward familiarity preferences whereas older infants showed a shift toward novelty preferences, as shown in Figure 2B. The difference between the age vs. preference shift correlations in the unilateral conditions (pooled) and bilateral condition was not significant (one-tailed permutation test on difference between correlations, *r*_unilateral_ – *r*_bilateral_ = 0.37, *p* = 0.127). However, exploratory analysis showed that these effects, based on summary preferences computed per child, were sensitive to coding and analysis parameters, as shown in Table 2.

**Table 2:** Robustness of effect size measures for Experiment 1 under varying analysis parameters.

Lookaway threshold (s)	Trials used	Exclude	*N*	Abs. diff.	*r*_bi_	*r*_uni_	*r*_diff_
1.00	4	–	49	0.07^*^	-0.05	0.32+	0.37
**0.50**	4	–	49	0.08^**^	-0.23	0.24	0.47+
**0.75**	4	–	49	0.07^*^	-0.20	0.27	0.48+
**1.50**	4	–	49	0.01	0.05	0.25	0.21
1.00	**6**	–	49	0.02	0.21	0.34+	0.13
1.00	4	**Invalid**	19	0.05	0.43	0.25	-0.18
1.00	4	**Short LT**	37	0.06^*^	-0.35	0.37+	0.72^*^
1.00	4	**Fussy/distracted**	38	0.06^*^	0.18	0.22	0.03


*The first row corresponds to the primary analysis. In each subsequent row, the parameter changed is shown in bold. Lookaway threshold: length of continuous lookaway required to end the LT measurement. Trials used: how many trials used to compute LT preferences in each test measurement – first four or all six. Exclude: *invalid* excluding participants with at least one invalid trial used; *short LT* excluding any participants with at least one trial used with a looking time under 1 s; *fussy/distracted* excluding participants who were fussing at the end of any trial used or where a distraction preceded the end of any trial used by under 1 s. *N*, number of included participants under these parameters. Abs. diff., mean absolute value of the change in preference in unilateral conditions minus mean absolute value of change in preference in bilateral condition. Significance assessed by one-tailed Wilcoxon rank sum test. *r*_*bi*_, Spearman rank correlation of age with preference shift toward the non-matching images, within bilateral condition. *r*_*uni*_, Spearman rank correlation of age with preference shift toward the non-matching images, within unilateral conditions (pooled). *r*_*diff*_: *r*_*uni*_ – *r*_*bi*_, significance assessed by one-tailed permutation test. ^+^*p* < 0.10; ^∗^*p* < 0.05; ^∗∗^*p* < 0.005.*

### Discussion

These findings suggest that infants shown identical shapes within a single hemifield more readily represented the relation between the two shapes, as evidenced by the greater absolute impact on their preference for looking at matching shapes at test. This is in contrast to the bilateral field advantage adults exhibit when comparing visual stimuli (Sereno and Kosslyn, 1991).

Abundant evidence shows that the quality of the representation constructed by an infant affects his or her preference for familiar stimuli. Younger infants, more complex stimuli, and less familiarization time can all lead to familiarity rather than novelty preferences (Rose et al., 1982; Hunter et al., 1983; Richards, 1997; Roder et al., 2000; Houston-Price and Nakai, 2004; Aslin, 2007; Kidd et al., 2012) A constant amount of familiarization, as performed in Experiment 1, could therefore yield a shift from familiarity to novelty preferences with age. (The age trend observed does not indicate a change in interhemispheric integration over the age range studied; rather, we are assuming that infants across this age range represent concepts similarly but express their familiarity differently with age.) We observed a possible shift in this direction in the unilateral conditions, in support of the interpretation that the increased variance in preference shifts was due to familiarity with the concept of “matching shapes.”

In contrast, the preference shifts between baseline and final testing of infants in the bilateral condition were not systematically related to age. The condition difference cannot be attributed simply to differences in peripheral position of or distance between the matching shapes, since we observed comparable trends when matching either for how peripheral or for how distant from each other the matching shapes were during familiarization.

This experiment must be regarded as purely exploratory, as the form of these results was unpredicted prior to initial analysis. However, the form of results informed future study design. Having found a possible effect on shifts in preference, but with direction potentially linked to quality of the familiarized representation, we next planned to look directly for differences in the relation between preference and age depending on lateralization of the familiarized stimuli.

## Experiment 2: Does 8 on the Left Plus 8 on the Right Look Like 16?

Even in the strongest interpretation of the results of Experiment 1, the question remains of whether infants in the bilateral condition simultaneously perceive two clear but incomparable shapes, or do not even sense that they are seeing multiple shapes. If the percept in question were *number* rather than similarity of shapes, we could distinguish these possibilities. Experiment 2 addresses perceptual integration more directly in the case of approximate numerical representations by familiarizing infants with 16 dots either all in one visual hemifield (16+0) or split evenly between the two hemifields (8+8). In this case, we can directly predict the representation that would result from a failure to integrate information: infants in the 8+8 condition will represent 8, whereas infants in the 16+0 condition will represent 16. Moving to the domain of approximate number has the additional advantage that the approximate number system is well-characterized in infancy, and parallel detection (Halberda et al., 2006) and local adaptation (Burr and Ross, 2008) are possible in adults, suggesting the existence of intermediate representations with retinotopic receptive fields. We expect that infants will be able to represent and distinguish these quantities since 6-month-old infants succeed in discriminating quantities with a 1:2 ratio (Xu et al., 2005) with sufficiently long presentations (Wood and Spelke, 2005), although the current task might be somewhat more challenging for infants given the shorter presentation times and the fact that the stimuli are presented in the periphery. In contrast to abstract same/different judgments of shapes, we have stronger reason to expect that numerosity will readily represented from brief presentations, as well as less reason to expect preferences based on individual stimuli (particular shapes vs. arrangements of dots), making results in this domain more straightforward to interpret.

In this study, we removed the baseline looking time measurement period to keep the test shorter and therefore infants less fussy, and to avoid any potential lasting effects of free viewing of baseline images on preferences. We also increased and expanded the age range slightly, to 10–19 months, given the potential complexity of a number-based task and in order to increase the probability of observing an age trend. We expect that younger infants may express what they experience during familiarization via familiarity preferences at test whereas older infants will show novelty preferences. That is, we expect that infants in the 8+8 condition will show a shift from preferring 8 (familiar) to 16 (novel) with age, whereas infants in the 16+0 condition will show a shift from preferring 16 (familiar) to 8 (novel) with age. However, depending on the overall novelty or familiarity preference displayed within the age group tested, and the rate of shift from familiarity to novelty preferences with age, we might observe either only this “cross” in preferences (an age × stimulus × condition interaction, without any overall condition difference in preferences) or only an overall condition difference in preferences. If all infants tested showed novelty preferences, for instance, we would see only a stimulus × condition interaction on looking times, corresponding to different overall preferences. We therefore conducted a planned test against the null hypothesis that *both* the condition × stimulus and age × stimulus × condition interaction terms were zero. An increase with age in the precision of numerical representation could explain age trends within either condition, but not such a condition difference.

There are minor methodological differences among studies 2 through 4 as we optimized the procedure, and results of the registered analyses are first presented separately; however, the structures are similar enough that the data have additionally been combined to yield overall estimates of effects.

### Methods

#### Participants

Forty-five infants (20 female) between 10 and 19 months of age (mean age 15.1 months) participated in this study. An additional 33 infants were excluded due to failure to complete the study (*n* = 18), experimenter error (*n* = 11), fussiness (*n* = 2), distraction (*n* = 1), or lack of at least one usable looking time trial per stimulus type (*n* = 1).

#### Procedure

The procedure consisted of (1) a familiarization period showing images of 16 dots and (2) a test of looking time to images of 16 and of 8 dots. The entire study lasted approximately 5 min, with a brief break (15 s) before the test period while parents were asked to close their eyes. Infants were assigned to bilateral (*n* = 22) or unilateral (*n* = 23) familiarization conditions. The procedure was identical to the one used in Experiment 1 except as follows.

##### Familiarization

**FIGURE 3:**
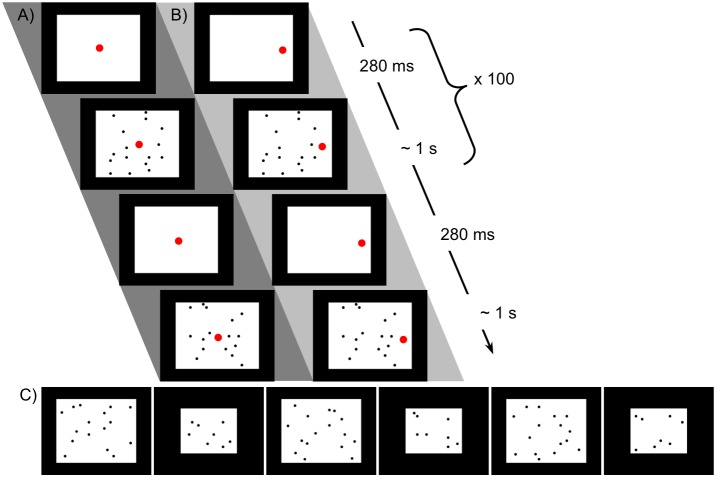
Stimuli used in Experiment for familiarization with 16 dots and preference test. **(A)** Schematic of procedure for familiarization with 16 dots, bilateral condition. The larger red dot indicates the position of the fixation video. **(B)** Unilateral condition familiarization, with all dots in one hemifield (the position of the fixation video at left or right was counterbalanced). The same images were used in the two conditions, with only the location of fixation altered. **(C)** Sequence of 16- and 8-dot images used for looking time tests in Experiment 2, in the order presented (left to right).

All infants were familiarized with images of 16 dots while looking at the fixation video on a monitor as in Experiment 1. In the ‘bilateral’ condition, eight dots appeared on one side of a central fixation video and eight dots on the other side. In the ‘unilateral’ condition, the same images were used but the fixation video was shifted to either the left or right side (counterbalanced and consistent within subjects). No images contained dots within the vertical segment containing the fixation video in any of its possible positions. No child saw any particular exemplar more than once, as there were 200 unique images available. The side of fixation (left or right) was counterbalanced between participants, so that each infant was familiarized with 16 dots in one consistent hemifield. Example sequences for each condition are shown in Figure 3A,B.

##### Looking time tests

Looking time to a series of six images, alternating between 16 and 8 dots as shown in the examples in Figure 3C, was measured as in Experiment 1. The dot size was the same as in familiarization images and the size of the white background was adjusted so that the densities of the 8- and 16-dot images were equal and equivalent to the density in the familiarization images. The order of presentation was consistent across all subjects to reduce variance associated with decreasing looking time over the six trials.

### Results

Data were coded from videotape as reported in Experiment 1. Analysis was conducted in MATLAB and marginal means plots were produced using emmeans (Lenth, 2018) in R (R Core Team, 2017); code is available in the OSF project repository. Due to the sensitivity of previous results to the details of analysis, we used a hierarchical linear model (linear mixed-effects model) to more robustly analyze data at the trial level, omitting individual trials with unreliable data. Individual trials meeting any of the following criteria were excluded from analysis: invalid trial (no valid lookaway and did not reach 30 s looking time); <1 s looking time; qualifying lookaway starts within 1 s after a distraction; child is fussy during qualifying lookaway; parent talks to the child during the looking time measurement. At least one usable trial per stimulus type was required for the child’s data to be included in analysis; one child was excluded for this reason.

Included looking time measurements were log-transformed as per Csibra et al. (2016). We modeled looking times per trial using the following hierarchical linear model:

LogLookingTime~Age*FamiliarizationType*TestImage+(TrialOrder+1|Child)

**FIGURE 4:**
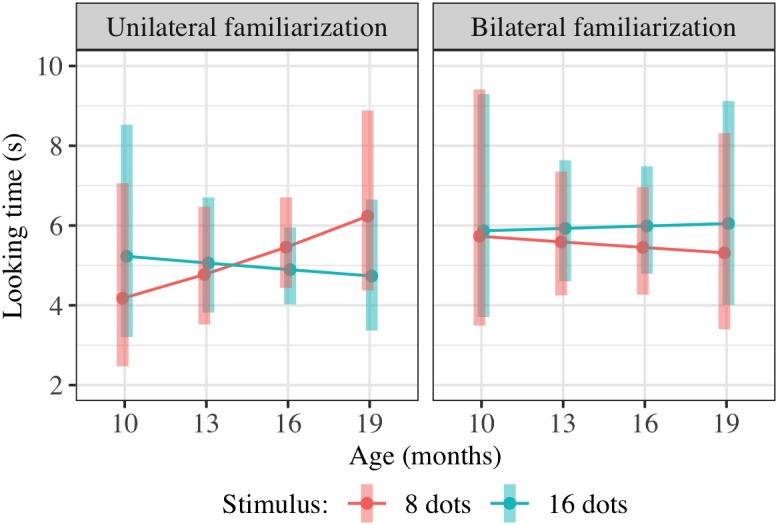
Interaction plot of estimated marginal means across combinations of familiarization condition and stimulus type over a set of reference ages spanning the tested age range, based on the linear mixed-effects model fitted for Experiment 2. Shaded error bars show 95% confidence intervals.

Age denotes the child’s age in months, centered on the mean age across measurements. FamiliarizationType denotes which form of familiarization the child received (1 = bilateral, 0 = unilateral) and TestImage which image type was being shown during this trial (1 = 16-dot image, 0 = 8-dot image). The reference condition, for interpretation of model coefficients, is therefore unilateral familiarization and viewing of an eight-dot image. The final term allows variation in mean looking times and slope of looking time with trial number (1–6) across children, including correlation between the random slopes and intercepts^1^. The results of this regression are shown in Table 3; estimated marginal means are shown in Figure 4. Degrees of freedom were estimated using the Satterthwaite approximation.

**Table 3:** Summary of hierarchical linear model of log-transformed test trial looking times in seconds for Experiments 2 and 4.

	Experiment 2					Experiment 4				
		
Variable	*B*	*SE B*	*df*	*t*	*p*	*B*	*SE B*	*df*	*t*	*p*
(Intercept)	0.72	0.04	100.3	16.32	0.000	0.61	0.04	123.7	16.65	0.000
Age	0.02	0.02	100.1	1.05	0.297	-0.01	0.02	124.9	-0.52	0.607
TestImage	-0.03	0.05	154.0	-0.56	0.578	-0.03	0.04	236.0	-0.82	0.413
FamiliarizationType	0.02	0.06	102.3	0.26	0.794	-0.06	0.05	126.3	-1.09	0.276
Age:TestImage	-0.02	0.02	154.5	-1.13	0.260	0.04	0.03	228.8	1.40	0.162
Age: FamiliarizationType	-0.02	0.03	99.7	-0.83	0.409	0.03	0.03	124.7	0.88	0.380
TestImage: FamiliarizationType	0.06	0.08	157.6	0.85	0.394	0.19	0.06	236.6	3.37	0.001
Age:TestImage: FamiliarizationType	0.03	0.03	155.2	0.93	0.355	-0.03	0.04	232.5	-0.71	0.476


*Data consist of 224 looking time measurements from 45 children in Experiment 2 and 337 looking time measurements from 66 children in Experiment 4, up to six measurements per child. Unstandardized coefficients and their standard errors are shown for fixed effects; degrees of freedom for *t*-tests are estimated via the Satterthwaite approximation. Age, age in months, centered on mean age. TestImage: 1 = 16 dots shown, 0 = 8 dots shown. FamiliarizationType: 1 = bilateral condition, 0 = unilateral condition.*

This model allowed us to test for the significance of *any effect* of condition on preference, including an overall difference in preference for 16-dot images between conditions in this age range or a difference in the interaction between age and preference between conditions. Although the three-way interaction was in the predicted direction (i.e., preference for 16-dot images increased more with age in the bilateral condition), the combined effect of FamiliarizationType ^∗^ TestImage and Age ^∗^ FamiliarizationType ^∗^ TestImage was not significant [*F*(2,156.4) = 0.79, *p* = 0.458]. That is, adding the two predictors FamiliarizationType ^∗^ TestImage and Age ^∗^ FamiliarizationType ^∗^ TestImage, corresponding to a predicted condition difference in preference and/or slope of preference with age, did not improve the predictive value of the model.

## Experiment 3

Given the broad age range, many factors beyond age that contribute to familiarity and novelty preferences, and the inconclusive results in Experiment 2, we designed Experiment 3 to look within a narrower age range exclusively for infants’ novelty preferences. We predicted that infants who had seen 16 dots unilaterally would treat 8 dots in free viewing on test as a novel stimulus but that infants who had seen 16 dots bilaterally would have represented seen something akin to “8 dots twice” and thus treat 16 dots at test as the novel stimulus. We also added a manipulation check condition in which infants saw eight dots unilaterally, to ensure that the basic prediction held, i.e., that infants familiarized to 8 dots would show a novelty preference for 16.

### Methods

#### Participants

Fifty-one infants (22 female) between 13 and 17 months of age (mean age 15.0 months) participated in this study. An additional 37 infants were excluded due to failure to complete the study (*n* = 11), experimenter error (*n* = 1), fussiness (*n* = 19), distraction (*n* = 1), parent interference (*n* = 4), or lack of at least one usable test trial per stimulus type (*n* = 1).

#### Procedure

**FIGURE 5:**
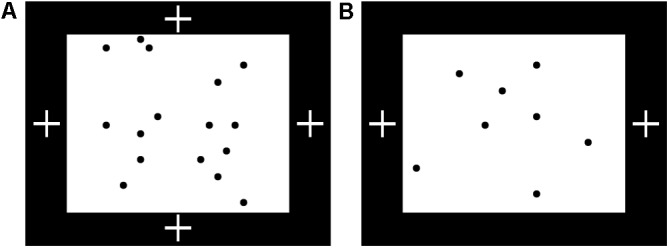
Familiarization setup for Experiment 3. **(A)** Possible positions of fixation video (white crosses) during familiarization with 16 dots in Experiment 3, with sample exemplar. The fixation video was shown at the top or bottom (bilateral condition) or left or right (unilateral-16 condition) of the screen, within a black border surrounding the images of 8 or 16 dots. **(B)** Position of fixation video during familiarization with eight dots, with sample exemplar.

The procedure was as in Experiment 2 except that the fixation video was moved from the white background area onto the black border of the screen, such that it was offset horizontally or vertically from the 16 dots to avoid a potential confound in which a fixation video in the midst of the dots might lead to visual grouping of the dots on either side. Infants were assigned to one of three conditions: bilateral (*n* = 15) or unilateral (*n* = 21) familiarization with 16 dots, or unilateral (*n* = 15) familiarization with eight dots, added as a manipulation check. Fixation video positions and sample familiarization exemplars are shown in Figure 5. The familiarization exemplars of 16 dots used in Experiment 2 were used for the ‘bilateral’ (*n* = 15) and ‘unilateral-16’ (*n* = 21) conditions. In the ‘unilateral-8’ condition (*n* = 15), only eight dots were presented during familiarization. Dots were the same size and spread across the same rectangular area as in the unilateral-16 condition, but less densely packed. There were always four dots in the left half of the image and four in the right half. The fixation video was shown at the left or right of the screen (counterbalanced) in the two unilateral conditions and at the top or bottom of the screen (counterbalanced) in the bilateral condition. The free-viewing test images were the same as in Experiment 2 except that the brightness of the background of the 16-dot images was reduced to equalize total luminance.

### Results

Data were coded from videotape as reported in Experiment 1. Potentially unreliable individual trials were excluded as in Experiment 2. We also omitted trials where the difference between looking time measurements calculated by categorizing “out of frame” periods (where the child’s eyes were not visible) as looking vs. not looking was at least 1 s; this criterion was not registered but arose during video coding, and affected five trials across two participants. (No participants in other studies were affected by applying the same criterion.) All participants had at least one usable trial per stimulus type.

Included looking time measurements were log-transformed and modeled using a hierarchical linear model as in Experiment 2, but without age effects:

LogLookingTime~FamiliarizationType*TestImage+(TrialOrder+1|Child)

**FIGURE 6:**
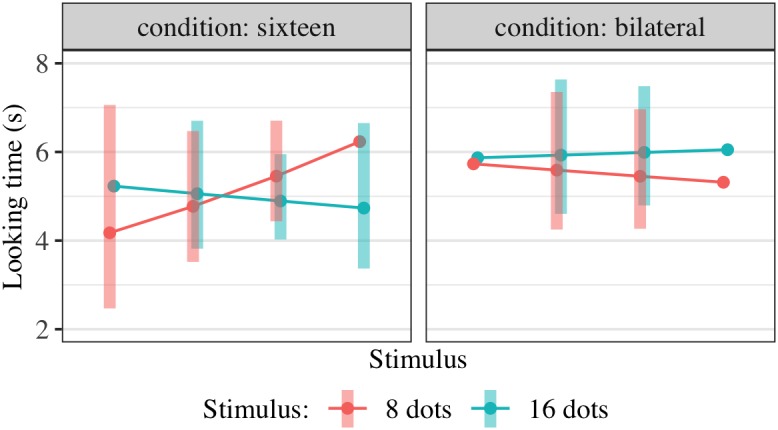
Interaction plot of estimated marginal means across combinations of familiarization condition and stimulus type, based on the linear mixed-effects model fitted for Experiment 3. Shaded error bars show 95% confidence intervals.

As in Experiment 2, FamiliarizationType was dummy-coded with unilateral-16 as the reference condition and dummy variables FamiliarizationType_Eight and FamiliarizationType_Bilateral representing the unilateral-8 and bilateral conditions, respectively. Results of this regression are shown in Table 4; estimated marginal means are shown in Figure 6. We checked for interactions between condition and stimulus, i.e., overall effects of condition on preference for 16 rather than 8 dots. We predicted that relative to those in the unilateral-16 condition, infants in unilateral-8 and bilateral-16 conditions would both perceive eight dots and therefore prefer 16-dot images more strongly, leading to positive TestImage × FamiliarizationType_Eight and TestImage × FamiliarizationType_Bilateral interactions. However, both interactions between were small and non-significant; there was no clear effect of condition on preference either between unilateral-16 and bilateral conditions [*t*(187.1) = 0.48, *p* = 0.629] or between unilateral-16 and unilateral-8 conditions [*t*(181.8) = -0.70, *p* = 0.483].

**Table 4:** Summary of hierarchical linear model of log-transformed test trial looking times in seconds for Experiment 3.

Variable	*B*	*SE B*	*df*	*t*	*p*
(Intercept)	0.55	0.05	82.3	11.82	0.000
TestImage	0.14	0.05	183.3	2.72	0.007
FamiliarizationType_Bilateral	0.03	0.07	87.8	0.46	0.650
FamiliarizationType_Eight	-0.09	0.07	91.3	-1.26	0.212
TestImage:FamiliarizationType_Bilateral	0.04	0.08	187.1	0.48	0.629
TestImage:FamiliarizationType_Eight	-0.06	0.08	181.8	-0.70	0.483


*Data consist of 254 looking time measurements from 51 children, up to six measurements per child. Unstandardized coefficients and their standard errors are shown for fixed effects; degrees of freedom for *t*-tests are estimated via the Satterthwaite approximation. TestImage: 1 = 16 dots shown, 0 = 8 dots shown. FamiliarizationType_Bilateral and FamiliarizationType_Eight are dummy variables representing the bilateral and unilateral-8 conditions, respectively, with unilateral-16 as the reference condition.*

## Experiment 4

Following the inconclusive results of Experiments 2 and 3, we planned a final, more conclusive internal replication of Experiment 2 with a larger sample size based on power analysis and the effect size detected in initial analysis of the dataset. We again used a broad age range and looked for a difference in familiarization-induced preference *and/or* its relation to age; to avoid potential concerns that the oldest children tested might be succeeding at integration and therefore responding on a different basis than the younger children, we capped the age range at 16 months. We again predicted that if infants perceived the bilateral displays as 8 rather than 16 dots, then we would observe condition differences in preference for 16 dots at test, a greater increase in preference for 16-dot images with age in the bilateral condition, or both.

### Methods

#### Participants

Sixty-six infants (32 female) between 10 and 16 months of age (mean age 13.3 months) participated in this study. An additional 22 infants were excluded due to failure to complete the study (*n* = 13), fussiness (*n* = 7), or lack of at least one usable looking time trial per stimulus type (*n* = 2).

#### Procedure

**FIGURE 7:**
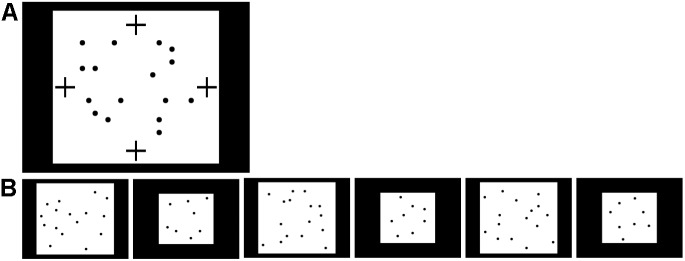
Stimuli used in Experiment 4. **(A)** Possible positions of fixation video (marked by black crosses) during familiarization with 16 dots in Experiment 4. The fixation video was shown at the top, bottom, left, or right of the screen, within the white rectangle containing the 16 dots. **(B)** Sequence of 16- and 8-dot images used for looking time tests in Experiment 4, in the order presented (left to right).

The procedure was as in Experiment 2 except that the fixation video was placed within the white image background of the familiarization images as shown in Figure 7A; it was not placed in the black border of the image in order to follow Experiment 2 more directly. Infants were assigned to bilateral (*n* = 32) or unilateral (*n* = 34) familiarization with 16 dots. A manipulation check as in Experiment 3 was initially planned but not pursued due to the rate of data collection. The fixation video was at the top or bottom of the image (counterbalanced) in the ‘bilateral’ condition and at the left or right of the image (counterbalanced) in the ‘unilateral’ condition. New familiarization exemplars were generated to accommodate this placement, with dots always offset horizontally and vertically from all possible fixation video locations; the same exemplars were used for both conditions. No child saw any particular exemplar more than once, as there were 150 unique images available. Test images, matched to the familiarization images for dot density, are shown in Figure 7B. In this experiment, a total of four fixation videos (two additional) were available to keep children’s attention.

### Results

Data were coded from videotape as reported in Experiment 1. Potentially unreliable individual trials were excluded as in Experiment 2. Included looking time measurements were log-transformed and modeled using a hierarchical linear model as in Experiment 2:

LogLookingTime~Age*FamiliarizationType*TestImage+(TrialOrder+1|Child)

**FIGURE 8:**
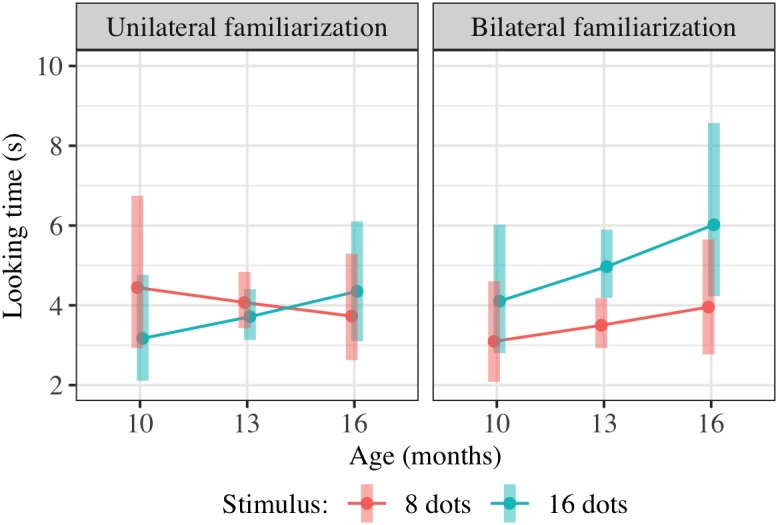
Interaction plot of estimated marginal means across combinations of familiarization condition and stimulus type over a set of reference ages spanning the tested age range, based on the linear mixed-effects model fitted for Experiment 4. Shaded error bars show 95% confidence intervals.

Results of this regression are shown in Table 3; estimated marginal means are shown in Figure 8. We checked for *any* effect of condition on preference as in Experiment 2, pooling the potential effects of condition on preference and of condition on preference-age slope together by comparing models with and without the two terms TestImage ^∗^ FamiliarizationType and TestImage ^∗^ FamiliarizationType ^∗^ Age. The combined effect of TestImage ^∗^ FamiliarizationType and TestImage ^∗^ FamiliarizationType ^∗^ Age terms was significant [*F*(2,234.5) = 5.91, *p* driven by a greater = 0.003]. These results show that the familiarization condition (bilateral or unilateral) did affect looking preferences at test, as predicted; the difference was driven by children showing an overall greater preference for 16-dot images in the bilateral condition, but not the unilateral condition.

### Combined Results of Approximate Number Studies (Experiments 2–4)

**FIGURE 9:**
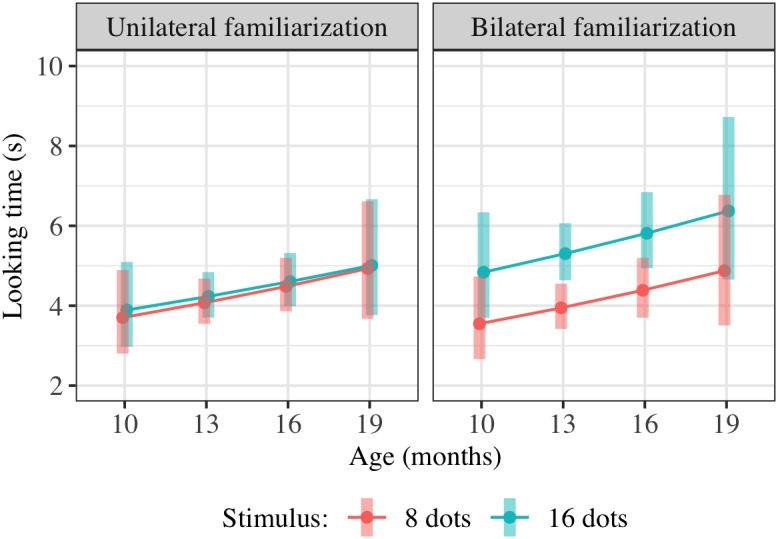
Interaction plot of estimated marginal means across combinations of familiarization condition and stimulus type over a set of reference ages spanning the tested age range, based on the linear mixed-effects model fitted using unilateral(-16) and bilateral familiarization data from Experiments 2, 3, and 4. Shaded error bars show 95% confidence intervals.

We conducted additional exploratory analysis to assess the overall condition effect (bilateral vs. unilateral presentation of 16 dots) across Experiments 2–4. Modeling all included trials across these experiments (739 trials from 147 children) using the same hierarchical linear model as in Experiments 2 and 4 also yields a significant combined effect of TestImage ^∗^ FamiliarizationType and TestImage ^∗^ FamiliarizationType ^∗^ Age terms [*F*(2,525.3) = 3.86, *p* = 0.022], driven by a greater preference for 16-dot images in the bilateral condition. Results of this regression are shown in Table 5; estimated marginal means for this model are shown in Figure 9. The effect was similar when excluding trials where parents peeked at stimuli [*F*(2,493.5) = 3.70, *p* = 0.025] and when using raw (rather than log-transformed) looking times [*F*(2,533.5) = 2.36, *p* = 0.095]. That is, the effect held when considering Experiments 2–4 together, and was not especially sensitive to the details of exclusion or analysis: bilateral familiarization with the same 16-dot images led to different looking preferences than unilateral familiarization.

**Table 5:** Summary of hierarchical linear model of log-transformed test trial looking times in seconds for pooled data from Experiments 2–4.

Variable	*B*	*SE B*	*df*	*t*	*p*
(Intercept)	0.63	0.02	276.0	25.13	0.000
Age	0.01	0.01	268.0	1.08	0.282
TestImage	0.01	0.03	518.3	0.54	0.591
FamiliarizationType	-0.01	0.04	283.8	-0.32	0.748
Age:TestImage	0.00	0.01	517.6	-0.12	0.906
Age: FamiliarizationType	0.00	0.02	271.8	0.08	0.935
TestImage: FamiliarizationType	0.11	0.04	527.1	2.78	0.006
Age:TestImage: FamiliarizationType	0.00	0.02	523.4	-0.02	0.986


*Data consist of 739 looking time measurements from 147 children, up to six measurements per child. Unstandardized coefficients and their standard errors are shown for fixed effects; degrees of freedom for *t*-tests are estimated via the Satterthwaite approximation. Age, age in months, centered on mean age. TestImage: 1 = 16 dots shown, 0 = 8 dots shown. FamiliarizationType: 1 = bilateral condition, 0 = unilateral condition.*

We further expanded the hierarchical linear model to include trials from all three conditions (unilateral-16, bilateral-16, and unilateral-8 presentations) across these studies, including participants in the unilateral-8 condition in Experiment 3 and participants in a comparable unilateral-8 condition in Experiment 4 which was abandoned early in the testing process due to the pace of data collection. Fourteen infants (eight females) between 11 and 16 months of age (mean age 13.7 months) participated in the unilateral-8 condition in Experiment 4; an additional 10 infants were excluded due to failure to complete the study (*n* = 6), fussiness (*n* = 2), or inattention (*n* = 2). Altogether, 889 trials from 176 children were included in this model. We did not observe a significant condition effect between the unilateral-16 and unilateral-8 conditions, i.e., an effect of TestImage ^∗^ FamiliarizationType_Eight and TestImage ^∗^ FamiliarizationType_Eight ^∗^ Age terms [*F*(2,597.9) = 1.28, *p* = 0.279]. These results mean that we failed to confirm the effect of the manipulation check. However, power to detect such an effect was decreased due to the smaller sample size in the unilateral-8 conditions, and the coefficients for FamiliarizationType_Eight and FamiliarizationType_Bilateral interactions (with TestImage and Age ^∗^ TestImage) were similar, with both showing an increased preference at test for 16-dot images.

Within the unilateral-16 conditions across Experiments 2–4, we did not observe any effects of the position (right or left) of the dots during familiarization on looking time preferences, indicating that our results were unlikely to be driven by hemispheric differences. Log-transformed looking times were modeled as:

LogLookingTime~Age*Side*TestImage+(TrialOrder+1|Child)

Side was dummy-coded with right-hemifield presentation (left-hemisphere processing) as the reference condition. No predictors were individually significant (*p* > 0.05), and the inclusion of presentation side as a predictor (i.e., the addition of the four terms involving side) did not significantly improve the model [*F*(4,149,9) = 1.30, *p* = 0.272].

### Discussion of Approximate Number Studies (Experiments 2–4)

These results provide support for the possibility that infants primarily represent the approximate numerosity within a single hemifield, rather than integrating across hemispheres. We predicted that the unilateral condition would familiarize infants with 16 dots, whereas the bilateral condition would familiarize them with 8 dots (twice). Depending on the overall quality of infants’ representations of the briefly presented exemplars, and the rate of change with age, this distinction could result in either overall differences in preference between conditions (condition ^∗^ stimulus interactions) or modulation of the age trend by condition (age ^∗^ condition ^∗^ stimulus interaction).

Only in the internal replication, Experiment 4, did the predicted condition effect rise to the level of significance; we observed a greater preference for 16 dots in the bilateral condition, consistent with a novelty preference. Encouragingly, this was the most recent study—using a difficult protocol which we ran more smoothly with practice—and had the largest sample size and lowest exclusion or dropout rate (25% in Experiment 4 vs. 42–43% in Experiments 2 and 3). This result also held when considering all three experiments together. However, the strength of this evidence must be evaluated in the context of the sequence of experiments conducted. That is, had Experiments 2 and 3 shown strong and robust effects, Experiment 4 might not have been conducted. Additionally, while the fixed trial order (16, 8, 16, 8, 16, 8) allowed minimizing noise due to order effects, it leaves open the possibility that apparent condition effects on preference for 16 dots could be due to condition effects on the rate of decrease in looking time across trials.

Because of infants’ demonstrated capacities for flexible combination of numerosities (McCrink and Wynn, 2004), we expect that merely representing single-hemifield numerosity as an initial step in computing total numerosity would not lead to the observed results. However, it is possible that the position of dots relative to fixation affects perception of grouping, such that infants are more likely to see “two groups of eight” in the bilateral condition and “one group of sixteen” in the unilateral condition. This could be addressed directly in further work using grouped test stimuli.

Additionally, infants might expect objects to be distributed uniformly throughout the available space, allowing for heuristic estimation of total numerosity from a unilateral percept without actual integration. This would reduce the expected differences in responses between unilateral and bilateral presentations of the same numerosities. Experimental designs requiring comparison rather than summation of numerosities (e.g., familiarizing with left < right) would in that case be expected to produce more robust effects; varying the placement of the fixation video for unilateral familiarization would allow direct assessment of the contribution of such expectations.

The possibility also remains that the more peripheral placement of the 16 dot exemplars in the unilateral condition affects infants’ perception of numerosity, or that movement into the periphery horizontally vs. vertically differentially affects numerosity judgments. Adults underestimate numerosities presented in the periphery (Valsecchi et al., 2013); if infants are subject to the same bias, there are two main possibilities for its impact. First, in the absence of any deficit in integration, they could perceive the more central 8+8 dots as more numerous than 16+0; this could explain a condition difference as observed in Experiment 4 as a global familiarity preference, with children in the bilateral condition preferring 16 more strongly because they were familiarized with a more clearly similar numerosity. Second, in the presence of a deficit in integration, the quantities perceived could be closer than expected (8 vs. 12, say, rather than 8 vs. 16), making an effect more difficult to observe. Further work using unilateral presentations would be necessary to disentangle these possibilities.

## General Discussion

These experiments suggest that even relatively late in infancy, children may not readily integrate visual information across hemispheres. We introduced a novel method for familiarizing infants with lateralized stimuli and demonstrated effects across two distinct domains: form perception and approximate number. Specifically, our results suggest that infants represented the congruency of shapes and the total number of dots when presented unilaterally but not bilaterally; when presented bilaterally, infants may have represented two shapes and two sets of eight dots without being able to integrate them into a single representation.

Several distinct challenges could in principle prevent infants from achieving representations of stimuli across the entire visual field. Our focus is on the integration of putative single-hemifield representations, but failure to perceive or remember the content of either hemifield could also explain these results—for instance, due to exclusive allocation of attention to one hemifield at a time. While stark hemispheric specialization could in principle also prevent interhemispheric integration, the absence of effects of right vs. left hemifield display on looking preferences within children shown 16 dots unilaterally during familiarization in Experiments 2–4 makes this explanation unlikely. Adult data likewise makes it unlikely that children are simply allocating attention to only one hemisphere at a time, unless there is substantial developmental change in the structure of attentional resources: adults are actually *more* able to maintain spatially separated attentional foci in different hemispheres (Malinowski et al., 2007) and may indeed use independent resources for object tracking in each hemifield (Alvarez and Cavanagh, 2004). Nevertheless, it will be important to establish whether both sides of the briefly presented stimuli are consistently seen and processed.

Assuming that interhemispheric integration is the primary hurdle infants face in our tasks, the question of how they eventually succeed remains. Is success based solely on a maturational trajectory as callosal axons are myelinated to speed information transfer, or is there genuine learning as infants map independent lateralized representations onto their counterparts in the opposite hemisphere? A purely maturational account would make the testable prediction that individual infants begin to succeed at the same age across a variety of domains, and with a variety of forms of integration (comparison, addition, etc.). Given the retinotopic organization *within* hemispheres as well, any particular delay in interhemispheric integration suggests at least a role for maturation as a prerequisite to efficient learning.

The current results must be regarded as exploratory, and further work will be required to more conclusively establish these effects – in particular, to clarify whether the preferences observed in the approximate number studies are genuine novelty preferences. Future work might also clarify the developmental trajectory of integration (when children begin to succeed), and better characterize the effects of familiarization during infancy given briefly presented, lateralized stimuli. Our testing arrangement at a children’s museum motivated us to test across as wide an age range as possible; however, this carries a corresponding reduction in the precision of effect estimates obtained. Due to noise inherent in looking time measurement, variation in infants’ initial underlying preferences, and variation in the quality of representation induced by familiarization, the difference between unilateral and bilateral familiarization would ideally be measured with substantially larger sample sizes, within-subjects designs, or comparisons across dimensions other than age. For instance, the amount of familiarization could be varied within a narrow age range, with a shift from familiarity to novelty expected with greater familiarization.

We have focused here on the challenge of interhemispheric integration in the visual domain, but in two respects this is potentially a special case of a more general problem. First, the same difficulty in interhemispheric transfer might apply not just to perceptual information processed in opposite hemispheres due to topographic mappings, but also to abilities that exhibit hemispheric specialization. In adults, a wide variety of abstract abilities such as language (for review, see Josse and Tzourio-Mazoyer, 2004), face recognition (e.g., Geffen et al., 1971; Ley and Bryden, 1979; Kanwisher et al., 1997), and statistical vs. perceptual causality judgments (Roser et al., 2005) are lateralized, with the corpus callosum mediating transfer of information from the more specialized hemisphere (Stephan et al., 2007). In many cases, hemispheric specialization is already present in infancy (e.g., Deruelle and de Schonen, 1991; Le Grand et al., 2003; Franklin et al., 2008). If young children struggle to integrate information across hemispheres, these lateralized abilities may be sequestered. For instance, early specialization of the left hemisphere for language (Mills et al., 1993; Holowka and Petitto, 2002) could make it difficult for children to respond to verbal questions about information processed in the right hemisphere, despite potentially being able to make other motor responses indicating understanding. Protracted development of verbal rather than implicit responses and surprising dependence of performance on task details are indeed observed in domains of theory of mind (Rhodes and Brandone, 2014) and intent-based moral judgment (Cushman et al., 2013), each of which has been suggested to be primarily a function of the right hemisphere (Siegal et al., 1996; Steckler et al., 2017).

Second, interhemispheric integration is also a special case of integrating the outputs of any brain systems that can process information independently – not just the two hemispheres. The challenge of interhemispheric integration, and the mechanisms by which it is eventually achieved, may therefore shed light on other computational puzzles facing young learners. In many cases, children fail to integrate relevant information from multiple specialized, encapsulated systems. Indeed, one of the strengths of the developmental approach to studying cognition is that it allows observation of separate foundational components of what appears in adulthood to be a single system. For example, doubly dissociated systems have been observed across domains of numerical cognition (Feigenson et al., 2004) and navigation (Lee and Spelke, 2010).

Finally, the extent to which infants integrate spatially distinct percepts and the mechanisms of developmental change have implications not just for cognition but for subjective experience. The current results suggest that infants may not experience a percept of seeing the left and right visual hemifields simultaneously, and that we all initially experience the world as split-brain patients. Analogous to the role of developmental approaches within the study of cognition, a developmental approach to studying consciousness would allow observation of typically developing infants rather than neurological patients to understand unity of experience.

## Author Contributions

KS designed the experiments, collected and analyzed data, and wrote the manuscript.

## Conflict of Interest Statement

The author declares that the research was conducted in the absence of any commercial or financial relationships that could be construed as a potential conflict of interest.

## Footnotes

1 Correlation between random slopes and intercepts were initially omitted from all models per the registered analysis plan, and were added during peer review. This change does not substantially impact fixed effect coefficients or *p*-values.
